# Bacteriophages Limit the Existence Conditions for Conjugative Plasmids

**DOI:** 10.1128/mBio.00586-15

**Published:** 2015-06-02

**Authors:** Ellie Harrison, A. Jamie Wood, Calvin Dytham, Jonathan W. Pitchford, Julie Truman, Andrew Spiers, Steve Paterson, Michael A. Brockhurst

**Affiliations:** ^a^ Department of Biology, University of York, York, United Kingdom; ^b^ Department of Mathematics, University of York, York, United Kingdom; ^c^ Institute of Integrative Biology, University of Liverpool, Liverpool, United Kingdom; ^d^ SIMBIOS Centre, Abertay University, Dundee, United Kingdom; University of California Irvine

## Abstract

Bacteriophages are a major cause of bacterial mortality and impose strong selection on natural bacterial populations, yet their effects on the dynamics of conjugative plasmids have rarely been tested. We combined experimental evolution, mathematical modeling, and individual-based simulations to explain how the ecological and population genetics effects of bacteriophages upon bacteria interact to determine the dynamics of conjugative plasmids and their persistence. The ecological effects of bacteriophages on bacteria are predicted to limit the existence conditions for conjugative plasmids, preventing persistence under weak selection for plasmid accessory traits. Experiments showed that phages drove faster extinction of plasmids in environments where the plasmid conferred no benefit, but they also revealed more complex effects of phages on plasmid dynamics under these conditions, specifically, the temporary maintenance of plasmids at fixation followed by rapid loss. We hypothesized that the population genetic effects of bacteriophages, specifically, selection for phage resistance mutations, may have caused this. Further mathematical modeling and individual-based simulations supported our hypothesis, showing that conjugative plasmids may hitchhike with phage resistance mutations in the bacterial chromosome.

## IMPORTANCE

Conjugative plasmids are infectious loops of DNA capable of transmitting DNA between bacterial cells and between species. Because plasmids often carry extra genes that allow bacteria to live in otherwise-inhospitable environments, their dynamics are central to understanding bacterial adaptive evolution. The plasmid-bacterium interaction has typically been studied in isolation, but in natural bacterial communities, bacteriophages, viruses that infect bacteria, are ubiquitous. Using experiments, mathematical models, and computer simulations we show that bacteriophages drive plasmid dynamics through their ecological and evolutionary effects on bacteria and ultimately limit the conditions allowing plasmid existence. These results advance our understanding of bacterial adaptation and show that bacteriophages could be used to select against plasmids carrying undesirable traits, such as antibiotic resistance.

## INTRODUCTION

As vectors of horizontal gene transfer, conjugative plasmids play an important role in bacterial adaptation by trafficking accessory traits of potential benefit to bacterial fitness between cells (1). The dynamics of conjugative plasmids are therefore important to the ecology and evolution of bacteria. Plasmid dynamics are derived, in part, from the balance between the costs of plasmid maintenance and the benefits conferred by plasmid accessory genes: traits carried on the plasmid which are beneficial to the host in particular environments. Theoretical conditions for the existence of plasmids within bacterial populations can be derived and their implications explored (2–6). Empirically, in environments where the benefits outweigh the costs, plasmids will persist near fixation through positive selection (7, 8). In the absence of positive selection, plasmids are expected to be purged from populations by purifying selection (9), unless plasmid decline is counteracted by sufficient conjugative transfer (10) and/or amelioration of the costs of maintenance (11, 12). Our understanding of the existence conditions for plasmids is limited, because bacteria-plasmid dynamics are typically studied in isolation, yet in nature bacteria are subject to interactions with other species. Particularly important among these are interactions with bacteriophages, which are ubiquitous and cooccur with bacteria in most environments, frequently outnumbering bacteria by as much as 100 to 1 (13). Lytic phages in particular have both ecological and population genetic effects on bacteria that are likely to affect plasmid persistence. First, phages are a major cause of bacterial mortality and thereby cause reductions in bacterial density (14, 15), which could reduce opportunities for plasmid conjugation and increase the probability of plasmid loss from the bacterial population. Second, phages impose strong selection pressures on natural bacterial populations (16, 17) and can drive recurrent selective sweeps of phage resistance mutations (18). These population genetics effects may impact plasmid dynamics in two non-mutually exclusive ways: plasmids may hitchhike on selective sweeps of phage resistance mutations (2), and/or there may be epistatic interactions between the costs of chromosomal phage resistance mutations and the cost of plasmid carriage (19).

We investigated how the ecological and population genetic effects of lytic bacteriophages on bacteria affect the persistence of conjugative plasmids by studying experimental evolution of the plant-associated soil bacterium *Pseudomonas fluorescens* SBW25 (20) and its naturally associated megaplasmid, pQBR103 (21), along with the lytic phage SBW25φ2 (18). pQBR103 carries a mercury resistance operon that allows the host bacterium to reduce mercuric ions to elemental mercury and thereby detoxify mercury-contaminated environments (22). We then used a combination of simple analytical mathematical models and individual-based evolutionary simulations to explain the observed dynamics. Mathematical models have been previously used to predict the basic behavior of plasmid population biology via conjugation and loss through segregation (2, 3). Individual-based modeling of bacterial population biology has been well explored (23, 24), but there are relatively few computationally explicit models of plasmid dynamics (6). Use of a tandem modeling approach allowed the more complex individual-based simulations to be benchmarked against simpler, general mathematical models that can be solved exactly, which is rarely attempted in interdisciplinary modeling (25).

## RESULTS

### Experimental evolution.

**FIG 1:**
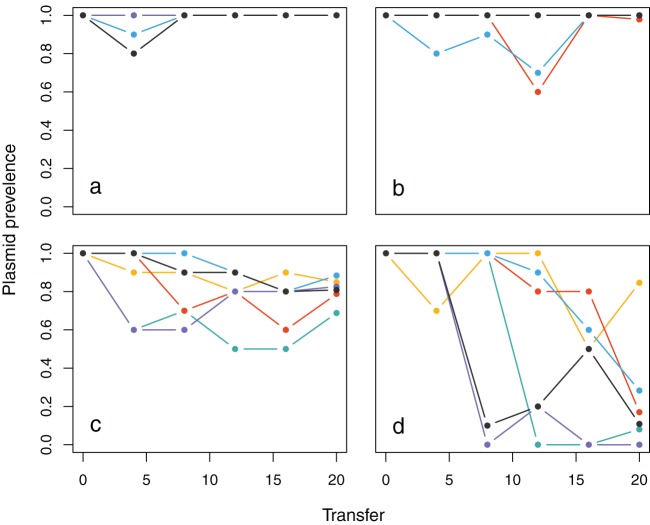
Prevalence of the pQBR103 plasmid over time in six replicate populations of each treatment. (a and b) The 32 µM HgCl_2_ environment without (a) or with (b) coevolving phages. (c and d) The 0 µM HgCl_2_ environment without (c) or with (d) coevolving phages. Colors distinguish individual replicate populations. Axes show the proportions of plasmid-carrying cells in the population (*y* axes) measured through time (*x* axes).

We first confirmed that plasmid carriage *per se* did not affect susceptibility of bacteria to phages (see Fig. S1 in the supplemental material). Next, six replicate populations were propagated in either the presence or absence of phages by serial transfer for c.130 bacterial generations in either mercury-free microcosms or microcosms supplemented with 32 µM HgCl_2_. At 0 µM HgCl_2_, pQBR103 imposes a net cost on bacterial fitness, whereas in the presence of mercuric ions plasmid carriage is beneficial (26). At 32 µM HgCl_2_, plasmids remained at or near fixation regardless of phage treatment, whereas at 0 µM HgCl_2_ plasmid prevalence declined over time (Fig. 1) (mercury × time; *z* = 4.44, *P* < 0.0001). Although phages reduced bacterial density in both mercury treatment groups (see Fig. S2 in the supplemental material) (*t*_16_ = −8.062, *P* < 0.0001), it was only with 0 µM HgCl_2_ that we observed clear differences in the dynamics of plasmid prevalence attributable to phages; in the absence of phages, plasmids gradually declined to intermediate frequencies in all populations, whereas in the presence of phages, plasmids remained at high frequencies for variable periods of time before rapidly declining to 0 or very low frequency in 5 out of 6 replicate populations. Phages therefore drove a more rapid loss of plasmids at 0 µM HgCl_2_ (phage versus time; *z* = 3.102, *P* = 0.002) and also led to higher variability in endpoint plasmid prevalence among replicate populations (*K*_1_ = 12.167, *P* = 0.0005; range of plasmid prevalence under 0 µM HgCl_2_ at transfer 12, without phage, 69 to 88%; with phage, 0 to 85%).

### Mathematical model.

To explore whether phage-induced mortality could explain these empirical dynamics, we developed a simple mathematical model. An existing mathematical model which captures the basic ecological dynamics (3) was used as a basis for the following pair of ordinary differential equations (ODEs), which describe the dynamics of a plasmid carrying accessory genes conferring mercury resistance:

(1) $$\frac{dF}{dt}=(\alpha F+\delta P)\left(1-\frac{F+P}{K}\right)-\gamma PF-\mu_{\text{bg}}F-\mu_{\text{phage}}F-\eta F$$

$$\frac{dP}{dt}=(\beta -\delta )P\left(1-\frac{F+P}{K}\right)+\gamma PF-\mu_{\text{bg}}P-\mu_{\text{phage}}P$$

where *F* and *P* are the concentrations of the plasmid-free and plasmid-carrying bacteria, respectively. Parameters α and β are their growth rates, and *K* is the carrying capacity. Transfer between types occurs through conjugation (where plasmids are passed to non-plasmid-carrying bacteria, parameterized by the rate γ) and by segregation (the spontaneous loss of a plasmid upon cell division, parameterized by δ). Effects of phages and mercuric ions are expressed as mortality effects, as phages require lysis of their host for replication and mercuric ions are bactericidal (27). All bacteria experience mortality via background effects (at the rate μ_bg_) and via exposure to phage (at rate μ_phage_), while bacteria in which the plasmid is absent suffer additional mortality via exposure to mercuric ions (at rate η). This model extends the work of Lili et al. (3) by explicitly including plasmid segregation effects and by separating the death terms to include both background effects, predation by phage and the poisonous effects of mercuric ions.

**FIG 2:**
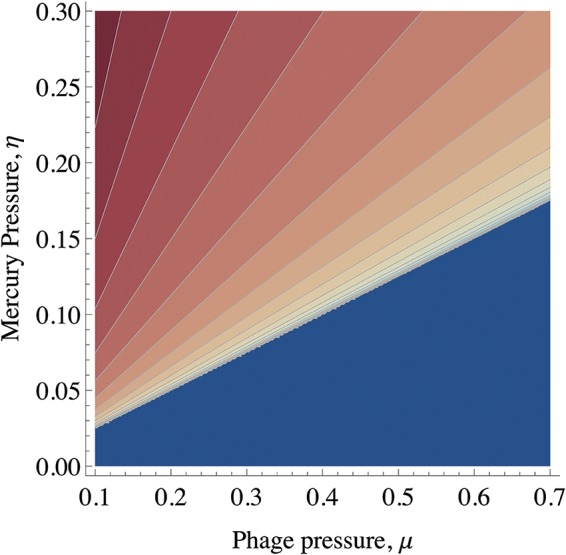
Plot of the stable fixed points of the mathematical model (equation set 1). The axes are the parameters μ and η, which are the mortalities associated with phage and mercury toxicity, respectively. Shading indicates the proportion of plasmid-carrying bacteria in the population. At high mercury concentrations and/or low phage mortality rates, plasmids are maintained at an interior fixed point, i.e., they coexist with plasmid-free cells (red; shading indicates the proportion of plasmid-containing cells, from high [dark] to low [light]). With a sufficiently low mercury concentration and under high phage pressure, plasmids are lost (blue). There is a line of transcritical bifurcations which separates these two regions. In the vicinity of the transcritical bifurcation, the convergence to the fixed point is slow enough that other factors (e.g., compensatory mutations [7, 12, 39–41]) are likely to occur prior to the model’s prediction of the loss or retention of the plasmid. The four corners of the plot correspond to the empirical treatments in Fig. 1 and the phase planes in Fig. 3. Parameter values are from Table 1.

**FIG 3:**
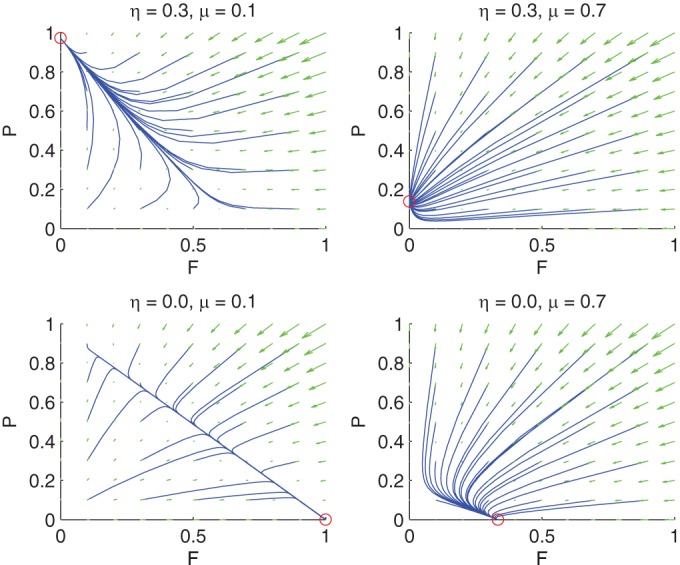
Mathematical phase planes for the population dynamics. Axes show total population values for plasmid-free (*F*; horizontal axes) and plasmid-containing (*P*; vertical axes) populations, scaled to the carrying capacity in the system without phage or mercury. The panels are arranged to correspond to both the overall diagram in the empirical data (Fig. 1) and the μ and η space (Fig. 2). The top panels show the 32 μM HgCl_2_ environment without phage (left; rapid fixation of *P*) and with phage (right; rapid fixation of *P*, reduced population density). The bottom panels show the HgCl_2_-free environment without phage (left; slow elimination of *P*) and with phage (right; rapid elimination of *P*, reduced population density). The blue curves show the trajectories from a grid of initial conditions, the green arrows indicate the global flow field, and the red circles show the stable fixed points to which trajectories are attracted.

The location and linear stability of the steady states of this model can be solved exactly, and the complete details are presented in the supplemental material. Figure 2 shows the behavior of the model (produced via equation set 1), illustrating how plasmid prevalence is expected to vary with phage and mercury pressure for parameter values describing the SBW25-pQBR103 interaction (Table 1). With high mercuric ion toxicity (η) or low mortality (μ), we observed retention of the plasmid; there was a stable interior fixed point (F*, P*) with a mixed population of plasmid-free cells and plasmid-carrying cells, respectively. In the absence of either mercuric ion toxicity (η) or phage mortality (μ_phage_), plasmids confer no advantage and are predicted to be lost from the system. Similarly, with high phage pressure and sufficiently low mercuric ion toxicity (η), the plasmid is lost completely from the system. These two regimes—where plasmids are either maintained or eliminated from the ecological system—are separated by a line of transcritical bifurcations given by:

(2) $$\eta =\frac{-\alpha \gamma K}{\beta -\delta -\gamma K}+\mu \frac{\alpha -\beta +\delta -\gamma K}{\beta -\delta -\gamma K}$$

which can be derived by analysis of either the fixed point position or the eigenvalues of the Jacobian matrices of the relevant fixed points. The phase plane dynamics of the model for parameters corresponding to the four experimental treatments are shown in Fig. 3. Exploration of the fixed parameters describing plasmid behavior (i.e., conjugation rate, segregation rate, and cost) demonstrated that this behavior is robust across a range of biologically realistic parameter values, with only the position of the fixed points (see Fig. S3 in the supplemental material) and, consequently, the line of transcritical bifurcations dependent on parameter space (see Fig. S4 in the supplemental material).

**TABLE 1:** Parameters for the mathematical model^a^

Parameter	Variable measured	Value	Source
α	Growth rate of a plasmid-free cell	1 h^−1^	Relative growth rates representing the cost of the plasmid in the plasmid-containing clones
β	Growth rate of a plasmid-carrying cell	0.8 h^−1^	
[1 − (μ_bg_/α)]κ	Measured carrying capacity of the system in phage- and mercury-free environments	7.3 × 10^9^ cells/ml	Estimated from CFU counts of phage-free, mercury-free populations, averaged through time (see also Fig. S2 in the supplemental material)
γ	Conjugation rate of the system	1.22 × 10^−14^ cell^−1^ h^−1^	Estimated within this system following the standard methods of Simonsen et al. (47)
μ_bg_	Background mortality rate	0.1 h^−1^	Consistent with information in reference 3; the exact value does not alter the qualitative dynamics
δ	Segregation rate	10^−4^ h^−1^	Segregation rate for a TOL plasmid in the *Pseudomonas* genus (51)
μ	Mortality due to phage, plus background mortality	0.7 h^−1^	Deduced from comparing the mathematical expressions at steady state with and without phage versus the empirical result that phage reduces population levels by 1/2 an order of magnitude (see Text S1 in the supplemental material for further details)
η	Mortality due to mercury	0.3 h^−1^	Estimated value; empirical results constrained to >0.174 (see Text S1 for further details)

^a^ Parameter values for the mathematical model (equation set 1) were estimated directly from empirical data where possible or drawn from the literature.

While in the presence of mercuric ions our purely ecological model captures the empirical dynamics of plasmids, the predicted stable points in the absence of mercuric ions do not explain the contrasting plasmid prevalence dynamics we observed. In the 0 µM HgCl_2_ phage-containing treatment, plasmids were initially maintained near fixation for various periods of time before being rapidly lost from the population (Fig. 1d). In contrast to the slow consistent decline in plasmid prevalence observed in the 0 µM HgCl_2_ phage-free treatment (Fig. 1c), these dynamics suggest that, under such conditions, the system displays bistability (in the mathematical sense of there being two stable points of attraction, plasmid fixation and plasmid loss). This dynamic behavior cannot be explained by our mathematical model (equation set 1), in which solutions follow a simple trajectory to the single stable fixed point in the system.

### Testing for selective sweeps of bacterial resistance to phages.

We hypothesized that this unexplained dynamic behavior could be due to the population genetic effects of bacteriophages on the bacterial populations, specifically by causing recurrent selective sweeps of phage resistance mutations on which plasmid-containing, or plasmid-free, backgrounds can hitchhike. To establish whether phages had caused selective sweeps of phage resistance mutations, we estimated the rate of phage resistance evolution at transfers 8, 12, and 16. To do this, we performed time-shift assays (28–30), whereby contemporary phage populations were tested against bacterial clones from four transfers in the past, the same transfer, and four transfers in the future. Positive slopes of bacterial phage resistance against time-shift would indicate that the operation of selective sweeps of mutations conferred resistance to bacteriophages over time. Consistent with this, we observed a highly significant positive relationship between time-shift and bacterial resistance (see Fig. S5 in the supplemental material) (time-shift × bacterial resistance, *t*_60_ = 4.81, *P* < 0.0001).

### Modified mathematical model and evolutionary simulations.

To explore whether this selective process could, in principle, explain the apparent bistability in plasmid prevalence, we made a simple modification to the mathematical model to incorporate the evolution of phage resistance. We assumed that resistance was most likely to evolve in the numerically dominant class and would therefore lead to positive frequency dependence in the mortality terms (31). Explicitly, we modified the phage-associated mortality rates in the original model (equation set 1) by introducing a weak dependence on the population fraction, leading to the following equation set for the populations:

(3) $$\frac{dF}{dt}=(\alpha F+\delta P)\left(1-\frac{F+P}{K}\right)-\gamma PF-\mu phage\left(1-\frac{\phi F}{F+P}\right)F-\mu {}_{bg}F-\eta F$$

$$\frac{dP}{dt}=(\beta -\delta )P\left(1-\frac{F+P}{K}\right)+\gamma PF-\mu_{phage}\left(1-\frac{\phi P}{F+P}\right)P-\mu_{bg}P$$

where the new parameter, ϕ, encodes the dependence of the mortality term. If ϕ is 0, then this model is identical to the equations above (equation set 1). This biologically motivated change leads to important changes in the behavior of the mathematical model. The system now exhibits the bistability suggested by the empirical data (see Fig. S6 in the supplemental material), such that for low mercuric ion levels in the presence of phage there can be two fixed points in the model with distinct basins of attraction. Complete details of the changes induced in the null cline structure can be found in Text S1 of the supplemental material.

**FIG 4:**
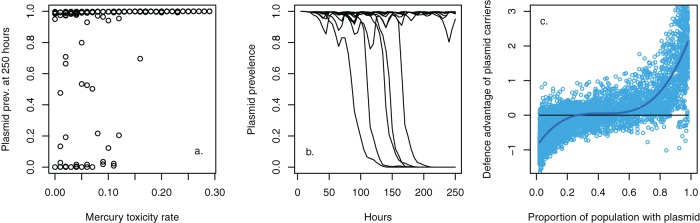
The individual-based model. The IBM captures the bistability of the system when phage is present. (a) The proportion of plasmid-containing cells after 250 h for 12 implementations across a range of mercury toxicity values, demonstrating that prevalence is predominantly fixed near either 1 or 0. (b) Plasmid frequencies through time for 12 replicate implementations under the poison = 0 condition (comparable to the results shown in Fig. 1d for the empirical data). (c) The positive frequency dependence appearing naturally in the IBM. Values denote the difference in the mean defense value for plasmid-carrying and plasmid-free cells, plotted against plasmid prevalence. Plasmid-carrying cells had a higher mean resistance to phage than plasmid-free cells when common and lower mean resistance when rare.

The simple mathematical models developed above provide a mathematical description of the observed empirical behavior, but such models can only coarsely approximate the complex evolutionary dynamics leading to the modified expressions in equation set 3. In particular, the assumption of positive frequency dependence and the interplay between ecology and evolution need careful justification. This is beyond the scope of the deterministic ODE models. We therefore developed an individual-based model (IBM) to simulate the evolutionary dynamics based upon an explicit two-locus model for chromosomal phage resistance and plasmid-carried mercury resistance. The IBM includes “arms race coevolution,” whereby both phage attack and bacterial defense traits evolve in a unidirectional “ratchet-like” manner, as observed in the empirical system (18), with a given mutation probability at each replication. Note that our IBM is designed to explicitly test the effects of allele frequency dynamics arising from coevolution on plasmid prevalence dynamics; therefore, we assume no costs of phage resistance or infectivity. The simulations are parameterized, where possible, using values directly estimated from the empirical system or, otherwise, using results of the ODE model to constrain parameter space. Consistent with our hypothesis, the establishment of a two-locus model with coevolutionary dynamics leads to positive frequency dependence and bistability in the IBM (Fig. 4), thereby justifying the inclusion of this modification to the mathematical model (equation set 3).

**FIG 5:**
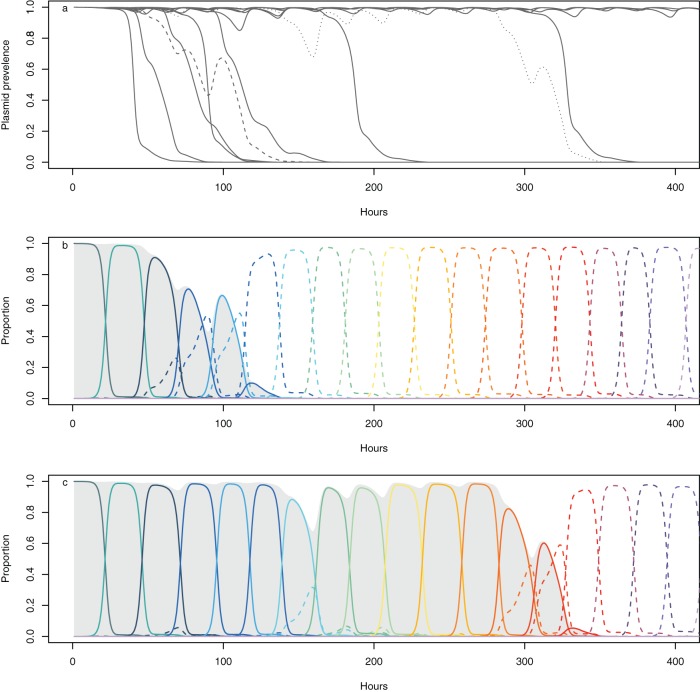
The dynamics of plasmid loss in the IBM simulation. (a) Plasmid dynamics in 12 iterations of the IBM. (b and c) Two iterations shown in detail (highlighted in panel a as coarse dashed [panel b] and fine dashed [panel c] lines) to demonstrate the link between phage resistance evolution and plasmid loss. In b and c, plasmid prevalence is shown by gray shading, and colored lines represent the frequencies of different phage resistance alleles present in the plasmid-containing (fixed) and plasmid-free (dashed) portions of the population. Plasmids are transiently maintained in the population by hitchhiking on sweeps of phage resistance mutations.

Figure 5 shows two examples of the dynamics within individual implementations of the IBM to illustrate how positive frequency dependence drives bistability. Plasmids are transiently maintained by hitchhiking to fixation with phage resistance mutations that arise in plasmid-containing backgrounds. However, plasmid-free cells are continuously generated through segregation and have a growth rate advantage over plasmid carriers, leading to repeated reinvasion and fluctuations in the ratio of plasmid-containing to plasmid-free cells. With higher frequencies of plasmid-free cells, the likelihood of successful phage resistance mutations occurring in this background is increased. Where this does occur, plasmid-free cells sweep to fixation, rapidly removing the plasmid from the population. In contrast to the deterministic mathematical model (equation set 3), the stochastic mutations and coevolutionary dynamics in the individual-based model enable the system to spontaneously jump from being dominated by plasmid-bearing bacteria to plasmids being lost from the system. As a consequence, we expect that the transient maintenance of the plasmid will lead to a large variability in the time-scale of plasmid loss, as observed in both the simulation (Fig. 4b) and with our empirical data (Fig. 1d).

### Testing for epistasis between costs of plasmid carriage and phage resistance mutations.

Finally, we sought to test whether negative epistasis between the cost of phage resistance and the cost of plasmid carriage may have contributed to accelerated plasmid loss by exacerbating the cost of plasmid carriage. Eight spontaneous phage resistance mutants were generated, and their competitive fitness was measured both with and without the plasmid (see Fig. S7 in the supplemental material). In the absence of the plasmid, phage resistance mutations imposed a fitness cost of between 11% (±1.8% standard error [SE]; *t*_2_ = −10.04, *P* = 0.01) and 37% (±12% SE; *t*_2_ = −4.42, *P* = 0.048). However, while the plasmid reduced host fitness by 14.5% (±1.2% SE; *t*_3.9_ = 7.85, *P* = 0.0015) in the ancestral, sensitive background, none of the plasmid-bearing phage resistance mutants had significantly lower fitness than their plasmid-free counterpart (*P* > 0.1). These data suggest that the costs of plasmid carriage and phage resistance mutations frequently demonstrate positive epistasis, which does not support the hypothesis that plasmid dynamics are driven by negative epistasis. Indeed, the observed positive epistasis would, if anything, promote the long-term maintenance of plasmids in the population, further supporting the idea that selective sweeps of phage resistance mutations best explain the different dynamics of plasmid prevalence observed at 0 µM HgCl_2_.

## DISCUSSION

Through their ecological and population genetics effects on bacterial populations, lytic bacteriophages limit the existence conditions for plasmids by accelerating their loss under weak or absent positive selection for accessory traits. This exacerbates the “plasmid paradox” (11, 32) by suggesting that plasmid persistence through horizontal transmission is less likely than previously predicted, due to the realistic condition of phage predation. There has been a strong thread of mathematical work on plasmid population biology, in particular, in determining the existence conditions for conjugative plasmids (3–5). However, these models have not considered the effects of bacteriophages on plasmid maintenance. Here, we have used simple models to show the ecological conditions under which plasmids can be lost from a system when under strong selection pressure from lytic bacteriophages. We have further demonstrated how the coevolution of phages and bacteria results in a positive frequency-dependent hitchhiking effect on plasmid populations by the use of individual-based simulation modeling. These simulations are calibrated using simpler deterministic mathematical models to give an unusual level of self-consistency within our approach. Furthermore, the population genetic dynamics derived from our simulation (see Fig. S8 in the supplemental material) are consistent with those already observed with this empirical system (33). The hierarchy of models so constructed enables us to understand the source of the underlying processes that give rise to each effect: the loss of the plasmid from the system is due to simple ecological dynamics; bistability emerges as a result of positive frequency dependence; recurrent sweeps of phage resistance mutations drive the transient maintenance of the plasmid, followed ultimately by irreversible loss.

Our findings suggest that phages are likely to impose strong indirect selection on conjugative plasmids. The ecological effects of lytic phages on the dynamics of plasmid prevalence are likely to apply generally, while the population genetics effects may be more restricted to phages that undergo persistent arms race coevolution with their bacterial hosts. Nevertheless, coevolutionary arms races have been reported across a taxonomically broad range of bacteria-phage associations (34, 35), suggesting that such dynamics are probably more common than has been previously suggested (36). Beyond the laboratory environment, changes to community and physical structure may alter the exact dynamics, for instance, conjugation rates can be several orders of magnitude higher in structured environments (37). Crucially, however, we have shown that the qualitative predictions of our model remain robust over a wide range of biologically plausible parameter values.

Given their ubiquity in natural communities and their widespread ecological effects on bacterial populations (14, 15), phages are potentially important drivers of bacteria-plasmid associations. Bacteria-phage coevolution has been shown to limit bacterial responses to non-phage-associated selective pressures (38). Thus, although bacterial compensatory evolution can help to stabilize plasmids (12, 39–41), this process may be less likely to occur in the presence of strong phage-imposed selection. By limiting the existence conditions for plasmids, we expect bacteriophages, in turn, to also alter selection on key plasmid traits. For example, phage-mediated plasmid purging is likely to select for the evolution of higher rates of conjugal transfer and greater amelioration of the physiological costs of carriage by plasmids, e.g., through gene loss (42) or reduced gene expression (8), to counteract higher rates of loss in the presence of phages. Our findings also have implications for bacterial evolution more broadly. Hitchhiking of conjugative plasmids on chromosomal mutations under strong selection extends the importance of hitchhiking in bacterial evolution from simply affecting other chromosomal traits in linkage (31, 43) to also affecting the dynamics of traits carried on mobile genetic elements, as originally hypothesized by Bergstrom and colleagues (2).

More rapid loss of plasmids in the time periods when or localities where plasmid accessory traits are not of benefit to the bacterial host suggests that accessory traits of potential benefit in other times and places may be lost to selection, reducing evolutionary potential. A case in point is accessory genes carrying antibiotic resistance: recent theory suggests that pulses of antibiotic selection can maintain conjugative resistance plasmids despite intervening periods of decline (44). Our data suggest that phages could dramatically shorten the interval between bouts of antibiotic selection that would otherwise support stable plasmid persistence. As such, our data potentially broaden the context for bacteriophage therapy against antibiotic resistance plasmids beyond the small subset of “male-specific” phages targeting plasmid-carried receptor genes (45) to coevolving lytic phages in general.

## MATERIALS AND METHODS

### Strains and culture conditions.

Experiments were conducted using the *Pseudomonas fluorescens* strain SBW25-ΩGm and its naturally associated megaplasmid, pQBR103 (21), along with the lytic bacteriophage SBW25φ2 (18). SBW25-ΩGm carries a gentamicin resistance marker (constructed following methods described by Koch et al. [46]), which allows the plasmid to be conjugated into this background following standard methods (47). We observed no difference in phage adsorption rate (measured as the reduction in phage density after 30 min of incubation with the host [48]) or efficiency of plating of SBW25φ2 between the plasmid-free and plasmid-containing strains, suggesting no direct effect of plasmid carriage on phage infection (see Fig. S1 in the supplemental material). All experiments were conducted in King’s B broth (KB) at 28°C in 30-ml microcosms containing 6 ml of medium with shaking at 180 rpm.

### Selection experiments.

Six replicate populations were founded for each of four treatments from independent clones of SBW25-ΩGm carrying pQBR103. Treatments comprised mercury-free (KB) and mercury-containing (KB with 32 µM HgCl_2_) environments in the presence or absence of bacteriophage in a full factorial design. Each population was established from 60 µl of an independent overnight culture (~10^7^ cells ml^−1^). Phage-containing treatments were inoculated with 6 µl of a 10^11^ phage/ml stock solution at the beginning of the experiment. Populations were propagated by serial transfer of 1% of culture to fresh medium every 48 h for a total of 20 transfers. Every 4 transfers, populations were screened for plasmid prevalence, and samples containing a final concentration of 20% glycerol were frozen at −80°C. Plasmid prevalence was estimated by colony PCR: initially, 10 colonies were screened using primers targeting the reductase gene *merA* (forward, 5′-TGCAAGACACCCCCTATTGGAC-3′, and reverse, 5′-TTCGGCGACCAGCTTGATGAAC-3′), which identify the presence of the mercury resistance operon, and primers identifying the putative origin of replication, *oriV* (forward, 5′-TGCCTAATCGTGTGTAATGTC-3′, and reverse, 5′-ACTCTGGCCTGCAAGTTTC-3′), which identify the presence of the plasmid. No instances of the loss of one target and retention of the other were observed during the experiment. Where fewer than 5 colonies were positive for the presence of the plasmid, a further 90 colonies were screened.

### Measuring the rate of phage resistance evolution in bacteria.

The rate of phage resistance evolution among bacteria was measured in the phage-containing, mercury-free treatment. Bacteriophage were isolated from each of the six replicate populations from transfers 8, 12, and 16 by filter sterilizing liquid cultures. Twenty bacterial clones were isolated from the same populations at transfers 4, 8, 12, 16, and 20. Each phage population was challenged with clones from its contemporary, sympatric bacterial population as well as clones of the same evolving lineage from 4 transfers in the past and 4 transfers in the future. Clones were streaked across lines of phage that had been previously dried onto a KB agar plate. Colonies showing inhibition of growth were scored as susceptible, while no inhibition of growth indicated resistance to phage.

### Estimating epistasis between costs of plasmid carriage and phage resistance.

To test for epistasis between the costs of phage resistance mutations and plasmid carriage, we first generated eight spontaneous phage resistance mutants in SBW25 by using a modified version of the fluctuation assay (49). Spontaneous mutants were used, as they represent an unbiased sampling of resistance mutations (i.e., have not been subject to selection) and are likely to be free of additional mutations unrelated to the bacterium-phage interaction. The ancestral strain, SBW25-ΩGm, was grown overnight and used to found 80 populations, each in 200 µl KB, in a 96-well plate at a starting density of ~2 × 10^6^ cells/ml. Simultaneously, eight phage-containing cultures were inoculated from freezer stocks. To ensure a range of phage resistance mutations, we collected phage from five evolved mercury-free populations as well as three ancestral bacteria-phage cocultures. Following overnight growth at 28°C, the 8 phage cultures were filter sterilized to provide 8 high-titer phage suspensions. Ten independent 200-µl cultures were then diluted to concentrations of 1:10 and 1:100 into each of the 8 phage suspensions, and 100 µl was plated onto KB agar. We then picked a single colony resistant to each of the phage suspensions, taken from the first of the 10 cultures where there was only one colony per plate.

The plasmid was conjugated into each of the phage-resistant mutants in addition to three independent cultures of phage-sensitive SBW25-ΩGm. Single mercury-resistant colonies for each of the 11 cultures were picked at random. These were confirmed to carry the plasmid by PCR and where appropriate to be phage resistant by streaking across phage. We then conducted competitive fitness assays for each of the 11 plasmid-free and 11 plasmid-containing isolates in triplicate. Briefly, overnight cultures of each strain were mixed at a 1:1 ratio with a *lacZ*-marked strain isogenic to the ancestor, inoculated into KB, and grown for 48 h. Samples were plated at 0 and 48 h onto KB supplemented with X-gal (5-bromo-4-chloro-3-indolyl-β-d-galactopyranoside), and relative fitness was calculated as the ratio of Malthusian parameters of competing strains (50).

### Statistical analyses.

All analyses were conducted with the R statistical package (R Foundation for Statistical Computing). Bacterial density and plasmid prevalence were analyzed using linear mixed-effects models. The former was analyzed using lme (package nlme) and the latter, which uses count data, with lmer (package lme4) and using a binomial error structure. In both cases, phage treatment was modeled as a fixed effect, with time and HgCl_2_ concentration as covariates and population as a random effect. The data were further investigated by separate analyses for each mercury treatment. The effects of phages in the mercury-free environment were analyzed separately in an lmer containing only these treatments. Differences in the rate of plasmid loss were determined by the interaction between phage treatment and time. Variance was tested using Bartlett’s *K* test. The cost of plasmid carriage was tested for significance in individual phage-resistant backgrounds by using Welch’s two-sample *t* test.

## SUPPLEMENTAL MATERIAL

Text S1  Mathematical models. Download Text S1, PDF file, 0.08 MB

Figure S1  SBW25φ2 phage infection dynamics on pQBR103-free (P−) and pQBR103-containing (P+) bacteria. (a) The phage adsorption rate was measured by estimating the drop in phage density after 30 min of incubation on each host. No significant difference was observed in the log_10_ adsorption rate into plasmid-containing compared to plasmid-free bacteria (*t*_2.8_ = 1.417, *P* = 0.258). (b) Efficiency of plating (EOP) onto the plasmid-containing strain was measured as the density of PFU when plated onto a lawn of plasmid-containing bacteria relative to the density when plated onto plasmid-free bacteria. The box plot shows the log_10_ plaque densities on both backgrounds. The mean EOP was not significantly different from 1 (mean EOP, 1.1 ± 0.302, *t*_8_ = 0.316, *P* = 0.76). Download Figure S1, EPS file, 0.01 MB

Figure S2  Bacterial density measured during the selection experiment. Colors denote independent replicate populations, corresponding to Fig. 3. (a and b) The 32 μM HgCl_2_ environment without (a) and with (b) coevolving phages. (c and d) The HgCl_2_-free environment without (c) and with (d) coevolving phages. Colors distinguish individual replicate populations. Download Figure S2, EPS file, 0.01 MB

Figure S3  Parameter variations in the phase plane. To test the dependence of the system on parameter variations, we evaluated the exact expression for the nontrivial fixed point when the three free paramters (β, γ, and δ) are chosen randomly (0.8 < β < 0.99; 0.0001 < γ < 0.1; 0.00001 < δ < 0.01) across four different mercury toxicity levels, η, under high phage pressure. Green points show stable points, red are unstable, and blue are stable points for the default parameter values used in the study. The position of the fixed point can vary, but there is no change in the qualitative structure of the plot. Only the position of the line of transcritical bifurcations changes; as a consequence, with decreasing mercury toxicity a greater proportion of fixed points fall beyond the line of transcritical bifurcations and the plasmid is completely lost. The position of the line of transcritical bifurcations is explored further in Fig. S4; the equation for this line of transcritical bifurcations is given in the supplemental text. Download Figure S3, PNG file, 0.04 MB

Figure S4  Exploration of the model across plasmid parameter space. The outcome of the model defined by equations 1 in the main text is shown, across a range of biologically plausible plasmid parameter values. The position of the line of transcritical bifurcations is plotted for segregation rates and conjugation rates 2 orders of magnitude above and below those approximated for the SBW25-pQBR103 system. The center plot therefore represents the parameters used in the model. Lines show the line of transcritical bifurcations at three levels of plasmid-containing growth rate (β): 0.7 (purple), 0.8 (red), and 0.9 (yellow). Red dots show the μ and η parameters sampled in the experiment. Despite movement in the position of the line, the qualitative predictions of the model are unchanged. Download Figure S4, PDF file, 0.1 MB

Figure S5  Bacteria-phage coevolution within the selection experiment. The rate of phage resistance evolution within the bacterial population was measured after 8, 12, and 16 transfers. Points show the mean proportion of resistant bacteria from past (−4 transfers), present, and future (+4 transfers) populations (left to right) when tested against contemporary phage populations. Means are averages of 6 replicate populations, with error bars showing ±1 SE. Download Figure S5, EPS file, 0 MB

Figure S6  Emergence of bistability in the ODE, including frequency dependence. Plots of the phase plane in the case where μ = 0.67 and η = 0.3 as the frequency dependent parameter is held below and above the fold bifurcation which gives rises to bistability. The point of bifurcation was computed to be fractionally larger than φ = 0.2; plots shown are for φ = 0.18 and φ = 0.25, respectively. Download Figure S6, EPS file, 0.1 MB

Figure S7  Fitness effects of phage resistance and plasmid carriage. Eight phage-resistant mutants were generated against a diverse set of phages (3 against the ancestral phage and 5 against phages taken from the evolved populations). The plasmid was then introduced to 3 sensitive and 8 resistant clones, and fitness was estimated based on competition with a marked plasmid-free ancestor. Sets of bars show mean fitness estimates (*n* = 3) for individual clones without (light) or with (dark) the plasmid. Shading shows phage sensitivity/resistance (sensitive, wide hash marks; resistant to ancestral phage, narrow hash marks; resistant to evolved phages, solid). Download Figure S7, PDF file, 0.03 MB

Figure S8  Comparison of outcomes of the ODE and individual-based model. The IBM run without evolutionary dynamics was able to reconstruct the behavioral portrait in the μ, η space predicted by the mathematical model (Fig. 2). Download Figure S8, EPS file, 0.3 MB

Figure S9  Oscillatory behavior in the individual-based model. The time series of the bacterial population over time reveals oscillations (left). These oscillations are correlated with the emergence of new phage resistance alleles that rapidly sweep through the population and lead to increased survival and a population increase. Download Figure S9, EPS file, 0.01 MB
