# Supplementary material for: Bacteriophages Limit the Existence Conditions for Conjugative Plasmids
Source: mBio. 2015 Jun 2;6(3):e00586-15. doi: 10.1128/mBio.00586-15 (PMC4453012; doi:10.1128/mBio.00586-15)
Supplement: Text S1 — Mathematical models. Download [file mbo003152350s1.pdf]

Bacteriophages limit the existence conditions for conjugative plasmids: Supplementary information

Ellie Harrison[1], A Jamie Wood[1,2], Calvin Dytham[1],Jon Pitchford[1,2], Julie Truman[3], Andrew Spiers[4], Steve Paterson[3], Michael A. Brockhurst[1]

[1] Department of Biology, The University of York, York, UK [2] Department of Mathematics, The University of York, York, UK [3] Institute of Integrative Biology, University of Liverpool, Liverpool, L69 7ZB, UK [4] SIMBIOSIS Centre, University of Abertay, Dundee, DD1 1HG, UK

Mathematical model without frequency dependence

We take model of Lili et al. [1] as our starting point; the authors develop a simple ordinary differential equation (ODE) model which captures the main features of our experimental system. The basic model [1] requires some modification for our purposes; in particular the segregative loss of plasmids is captured through spontaneous loss rather than transfer between types. This must be explicit in our model so as to capture the loss from mercury poisoning and from phage.

Our basic system therefore has the following governing equations:

$$\begin{aligned}\frac{d[F]}{dt} &= (\alpha[F] + \delta[P])(1 - \frac{[F]+[P]}{K}) - \gamma[F][P] - (\mu+\eta)[F] \\ \frac{d[P]}{dt} &= (\beta - \delta)[P](1 - \frac{[F]+[P]}{K}) + \gamma[F][P] - \mu[P]\end{aligned} \qquad (1)$$

where $[F]$ and $[P]$ are the concentrations of the plasmid free (F) and plasmid carrying (P) bacteria respectively, α and β are their growth rates and K is the carrying capacity. Transfer between types occurs through conjugation – where plasmids are passed to non carrying bacteria (the process $P+F \to P+P$) – parametrised by the rate γ and by segregation – the spontaneous loss of a plasmid upon cell division – parametrised by δ. Bacterial mortality occurs explicitly in three forms, exposure to mercury without the protection of the genes carried on the plasmid at a rate η, through background effects μ_{bg} and exposure to phage μ_{phage} so that death occurs at the rate $\mu = \mu_{bg} + \mu_{phage}$.

Parameters

The basic model is of simple enough form that its steady states may be solved exactly, and this allows our experimental outcomes to be used in order to arrive at representative parameter values. We note however that in this structurally simple model there is a wide range of parameter

values where the qualitative behaviour of the system is unchanged. We assume that the growth rate or turnover rate of a plasmid free cell, α is 1 hr^{-1}. We assume that the penalty for having a plasmid means the rate for a plasmid carrying cell, β is 0.8 hr^{-1}, based on the largest cost estimate from the empirical results (Fig S5). We assume a background mortality rate of $\mu_{background} = 0.1$ to ensure a turnover of cells in the system. The carrying capacity of the system is estimated to be 7.3×10^9 cells ml^{-1} in the phage and mercury free environments, derived from CFU counts averaged through time (see Fig. S2); in the model this corresponds to an assumption that $(1 - \frac{\mu_{bg}}{\alpha})K = 7.3 \times 10^9$ cells ml^{-1}.

The conjugation rate of the system, γ has been estimated to be between 10^{-14} ml $cell^{-1}$ min^{-1} to 6×10^{-13} ml $cell^{-1}$ hr^{-1} [3]. In spatially structured systems rates of up to 3 orders of magnitude higher have been reported [4].

We estimated conjugation rate of the pQBR103 plasmid in P. fluorescens to be $1.22 \times 10^{-14} \pm 4.69 \times 10^{-15}$ ml $cell^{-1}$ hr^{-1} following standard methods in Simonsen et al [5]. This is comparable to reported literature values which suggest that γK takes a value in the range of 4.3×10^{-3} ml $cell^{-1}$ hr^{-1} to 6×10^{-6} ml $cell^{-1}$ hr^{-1}, with the highest plausible biological value in structured populations of approximately 4. The structure of phase portrait is only weakly dependent on this choice as long as both $\delta \ll \alpha - \beta$ and $\gamma K \ll \alpha - \beta$; this is true across the full range of possible parameter values suggested from the empirical data. The mathematical literature suggests a wide spread of values for γK – Bergstrom et al [2] take values ranging from 5×10^-3 to 12 and Lili et al. [1] use values ranging between 0.3 and 3 (all in units of hr^{-1} where K is scaled to 1). The value for the segregation rate, δ for a TOL plasmid in the pseudomonas genus has been estimated to be 10^{-4} hr^{-1} [6].

In the case of only phage present we can estimate the value of μ_{phage}. The observed carrying capacity of the system is noted to be reduced by half an order of magnitude in the prescence of the phage. We can use this observation to approximate μ, by comparing $1 - \frac{\mu_{background}}{\alpha} \approx 3 \times (1 - \frac{\mu_{bg} + \mu_{phage}}{\alpha})$ giving $\mu_{phage} \approx 0.6$.

To estimate η is not a trivial task due to the nonlinearities in the expression for the interior fixed point i.e. the solution of the quadratic given for the fixed point of (1) given in the main text. The model predicts a lower value for the plasmid retaining population than is found empirically, which is probably due to the effect of mercury detoxification during extended growth. For the value of μ predicted by the above anaysis we predict the transition from retention to loss to happen at the mercury pressure of $\eta \approx 0.174$. Empirical evidence reveals that at $8, 16$ and 24μ M of mercury plasmids still persist and the population is largely unchanged; this is consistent with model but means it is difficult to arrive at a point estimate for η. We take a representative value of $\eta = 0.3$.

Analysis

For the set of equations given the behaviour of the system is as follows: There are 4 fixed points in the system: a trivial point at $(F = 0, P = 0)$ which is unstable for $\eta + \mu < 1$ and a saddle if $\eta + \mu > 1$; a plasmid loss point at $(F = 1 - \frac{\mu}{\alpha}, P = 0)$; and two other non-trivial fixed points which are either both unphysical (i.e. involving negative populations), or one is unphysical and the other is strictly positive. By analysing the existence and stability of these fixed points we can conclude that the system is in one of two physical configurations: EITHER the plasmid loss

point is stable and both non-trivial points are unphysical and unstable, OR the plasmid loss point is a saddle, in which case one of the non-trivial points is in the interior and stable and plasmids are retained in a mixed population. The boundary between these two regions is a line of transcritical bifurcations given by the line

$$\eta = -\frac{\alpha\gamma K}{\beta - \delta - \gamma K} + \frac{\alpha - \beta + \delta + \gamma K}{\beta - \delta - \gamma K}\mu, \tag{2}$$

which is found by analysis of either the fixed point position or the eigenvalues of the Jacobian matrices of the relevant fixed points. The line of transcritical bifurcations given by (2) is the only structurally important feature in the phase diagram, so this allows us to examine systematically how the biological parameters affect the system-level behaviour. Provided $\delta + \gamma K$ is less than $\alpha - \beta$ (the relative cost of carrying the plasmid), the slope in η-μ space of the line defined by (2) is unaffected by δ and γK and the gradient tends to $\frac{\alpha-\beta}{\beta}$ as $\delta + \gamma K \longrightarrow 0$.

The intercept of this line with axis will shift slowly towards zero from below as $\delta + \gamma K \longrightarrow 0$ – this means that the fixed point when $(\eta = 0, \mu = 0)$ is predicted by this model to always have plasmids retained. However, the dynamics in the region close to the line in (2) become very slow. Empirical estimates for compensatory mutations suggest that the cost of plasmids is ameliorated after 50-100 generations [13]. This corresponds to the eigenvalues of Jacobian matrix having a magnitude of less than ≈ 0.02, i.e. $\frac{1}{50}$, which defines a region symmetrically around the line of transcritical bifurcations where the unmodelled evolutionary dynamics make the output of the ecological model of limited value.

For all parameter values there is value of η below which plasmids are lost from the system entirely, and this value varies linearly with the phage pressure μ_{phage}.

Lili et al. [1] report in a similar system the possibility of the interior point becoming a stable focus with the potential to drive oscillatory behaviour. We find that, in our system, the conjugation rate used is achieve this is very high when compared to biologically plausible values for bacteria in solution. Explicitly, the value for the conjugation and segregation rates required are unrealistically high - the segregation rate needs to be around 3 orders of magnitude higher and the conjugation rate approximately 1 order of magnitude higher than the values we adopt. Therefore we do not consider this oscillatory possibility any further.

Mathematical model with frequency dependence

The data suggest that in the case where the plasmid is lost there exists a bistablity between plasmid loss and plasmid retention. The analysis above shows that the initial model (1) cannot exhibit this behaviour. Moreover, it does not include the selective sweeps which endow populations with phage resistence in a positively frequency dependent way, a phenomenon which is seen explicitly in experiments which track the population numbers [10]. As an approximation to this effect we include a first order approximation to positive frequency dependence in the phage

death rate, which results in a new model with governing equations

$$\frac{d[F]}{dt} = (\alpha[F] + \delta[P])(1 - \frac{[F]+[P]}{K}) - \gamma[F][P] - (\mu_{phage}\left(1 - \phi\frac{[F]}{[P]+[F]}\right) + \mu_{bg} + \eta)[F]$$
$$\frac{d[P]}{dt} = (\beta - \delta)[P](1 - \frac{[F]+[P]}{K}) + \gamma[F][P] - \mu_{phage}\left(1 - \phi\frac{[P]}{[P]+[F]}\right)[P] - \mu_{bg}[P] \quad (3)$$

where ϕ is a new variable which describes the strength of the frequency dependent effect (so that $\phi = 0$ corresponds to the original model (1).

Analysis

This system can exhibit more complicated bifurcational behaviour, with multiple branches to the nullclines. From the point of view of the biological system we are concerned with one key one aspect, namely the retention of the plasmid and the possibility of bistability. In the region where the plasmid is lost in the original model (1), if ϕ is increased from 0 a fold bifurcation occurs (at $\phi \approx 0.242$ for the parameters described here). The results in the emergence of a new stable point with high plasmid numbers which is separated from the plasmid loss fixed point by a saddle. This creates two distinct basins of attraction, resulting in bistablity between plasmid loss and plasmid retention as indicated in the experimental data.

Individual based model

The mathematical models enable us to understand the ecological dynamics, but not the details of the evolutionary trajectories induced by selective sweeps of new mutations through the population. To uncover this and explore the origin of the frequency dependent terms, we construct an individual event based model of the same system.

We identify four types of event within our individual based model: encounter with phage; death from other sources; conjugation and fission, with possible events occurring in series and time moving on incrementally after each event (for n bacteria time increments are drawn from a negative exponential distribution with a mean of $1/4n$). An individual bacterium is selected at random along with an event type. Following [12] we use a rejection method so events that do not occur still result in a time increment. Event probabilities are scaled so that there are $4n$ events in an hour. Encounters with phage occur with a variable probability. Probability of death from other sources is 0.1, plus the mortality effect of environmental mercury on plasmid-free cells. On conjugation a second bacterium is selected at random. If one bacterium carries a plasmid and the other does not there is a variable probability (usually 0.004) that the plasmid free cell gains a plasmid. Fission occurs using a logistic probability n/K for plasmid free cells and $0.8(n/K)$ for plasmid carrying cells. In these simulations K is fixed at 1000000. The derived cell will retain the plasmid status of its parent, but there is a 0.0001 probability of segregation leading to loss of plasmid. Each realisation runs for 250 hours. We varied phage pressure from 0 to 0.6 in steps of 0.02 and mortality from environmental mercury levels from 0 to 0.4 in steps of 0.02. The evolutionary dynamics associated with this model were initially turned off and compared to the results from the mathematical model (Fig S6). Our event based model is thus able to capture

all the results seen in the mathematical model. Results from this simulation are qualitatively identical to those from (3).

Evolution

Evolutionary dynamics were added to the IBM through the addition of ratchet type offence and defence properties. Phage are able to increase there attack potential through a series of stages and able to attack and kill bacteria of the same or equal defence level. Bacteria evolve defence against this attack in a similar way. Both attack and defence evolve through spontaneous mutation and are heritable opun growth. This one-dimensional representation of attack-defence phenotype space is justified with reference to the tracked mutations in the phage where all beneficial mutations occur in the LPS binding mechanisms.

When a bacterium encounters a phage the cell will only die if its defence is matched or exceeded by the phages attack. At the start of each simulation all attacks and defences are set to the same value. We assume a pool of phage with a static size of 1000000, and on cell lysis 20 new phage with the same attack strategy as their parent replace existing phage. There is a probability of ratchet mutation of defence on bacterial cell division and of phage attack on production of new phage at cell lysis.

This mechanism leads to bistability in the dynamics as anticipated from the mathematical model and importantly was can establish that the evolutionary dynamics gives rise to frequency dependence of the expected form.

Oscillatory Behaviour

The evolutionary behaviour leads to oscilliations in the bacterial population which are correlated with the emergence of new resistence alleles within the population (Fig S7). These oscillations are not predicted within either of the differential equation models proposed through the explicit emergence of a limit cycle nor through the emergence of stochastic resonance [9] as we have eliminated the possibility of a stable focus in this parameter regime. The existence of oscillations has been predicted empirically by Buckling et al. [10] who demonstrated the emergence of new alleles on a timescale of ≈ 50 hours. With the parameters established here we are able to show that a mutation rate of approximately 10^{-6} gives rise to selective sweeps on a similiar time scale. This value for the mutation rate compares well with experimental evidence [11].

References

[1] Lili LN, Britton NF, Feil EJ (2007). The persistence of parasitic plasmids.Genetics. 2007 Sep;177(1):399-405.

[2] Bergstrom C T, Lipsitch M. and Levin B. R. (2000). Natural selection, infectious transfer and the existence conditions for bacterial plasmids. Genetics. 2000 Aug;155(4):1505-1519.

[3] B F Smets, B E Rittmann and D A Stahl (1993). The specific growth rate of Pseudomonas putida PAW1 influences the conjugal transfer rate of the TOL plasmid. Appl. Environ. Microbiol. 1993, 59(10):3430.

[4] Cristina Lagido, Ian J Wilson, L.Anne Glover, Jim I Prosser (2003). A model for bacterial conjugal gene transfer on solid surfaces. FEMS Microbiology Ecology. Volume 44, Issue 1, pages 6778.

[5] Simonsen L, Gordon DM, Stewart FM, & Levin BR (1990) Estimating the rate of plasmid transfer - an end-point method. Journal of General Microbiology 136:2319-2325.

[6] Wouter A. Duetz d Johan G. van Andel (1991). Stability of TOL plasmid pWWO in Pseudomonas putida mt-2 under non-selective conditions in continuous culture. Journal of General Microbiology (1991), 137, 1369-1374

[7] Stephen M. Krone, Ruinan Lu, Randal Fox, Haruo Suzuki, and Eva M. Top (2007). Modelling the spatial dynamics of plasmid transfer and persistence. Microbiology. 2007 August ; 153(Pt 8): 28032816. doi:10.1099/mic.0.2006/004531-0.

[8] Molin, S (Molin, S); Tolker-Nielsen, T (Tolker-Nielsen, T) (2003). Gene transfer occurs with enhanced efficiency in biofilms and induces enhanced stabilisation of the biofilm structure. CURRENT OPINION IN BIOTECHNOLOGY 14(3) 255-261 DOI: 10.1016/S0958-1669(03)00036-3

[9] McKane, AJ; Nagy, JD; Newman, TJ; Stefanini, MO (2007). Amplified biochemical oscillations in cellular systems. Journal of Statistical Physics. 2007 July; 128: 165-191. DOI:10.1007/s10955-006-9221-9

[10] Alex R. Hall, Pauline D. Scanlan, Andrew D. Morgan, Angus Buckling. Hostparasite coevolutionary arms races give way to fluctuating selection (2011). Ecology Letters. Volume 14, Issue 7, pages 635642, July 2011

[11] Csaba Pal1, Mara D. Maci, Antonio Oliver, Ira Schachar & Angus Buckling (2007). Coevolution with viruses drives the evolution of bacterial mutation rates. Nature 450, 1079-1081 doi:10.1038/nature06350

[12] Allen & Dytham (2009) An efficient method for stochastic simulation of biological populations in continuous time. Biosystems. 2009 Oct;98(1):37-42. doi: 10.1016/j.biosystems.2009.07.003. Epub 2009 Jul 14.

[13] A. San Millan et al. (2014) Positive selection and compensatory adaptation interact to stabilize non-transmissible plasmids. Nature Communications 5, 5208 doi:10.1038/ncomms6208
