# Supplementary figures and images for: Bacteriophages Limit the Existence Conditions for Conjugative Plasmids
Source: mBio. 2015 Jun 2;6(3):e00586-15. doi: 10.1128/mBio.00586-15 (PMC4453012; doi:10.1128/mBio.00586-15)

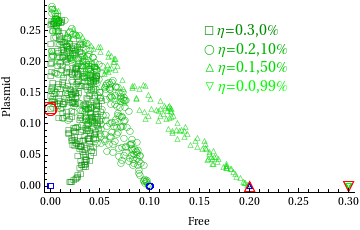

Supplement: Figure S3 — Parameter variations in the phase plane. To test the dependence of the system on parameter variations, we evaluated the exact expression for the nontrivial fixed point when the three free paramters (β, γ, and δ) are chosen randomly (0.8 < β < 0.99; 0.0001 < γ < 0.1; 0.00001 < δ < 0.01) across four different mercury toxicity levels, η, under high phage pressure. Green points show stable points, red are unstable, and blue are stable points for the default parameter values used in the study. The position of the fixed point can vary, but there is no change in the qualitative structure of the plot. Only the position of the line of transcritical bifurcations changes; as a consequence, with decreasing mercury toxicity a greater proportion of fixed points fall beyond the line of transcritical bifurcations and the plasmid is completely lost. The position of the line of transcritical bifurcations is explored further in Fig. S4; the equation for this line of transcritical bifurcations is given in the supplemental text. Download [file mbo003152350sf3.png]

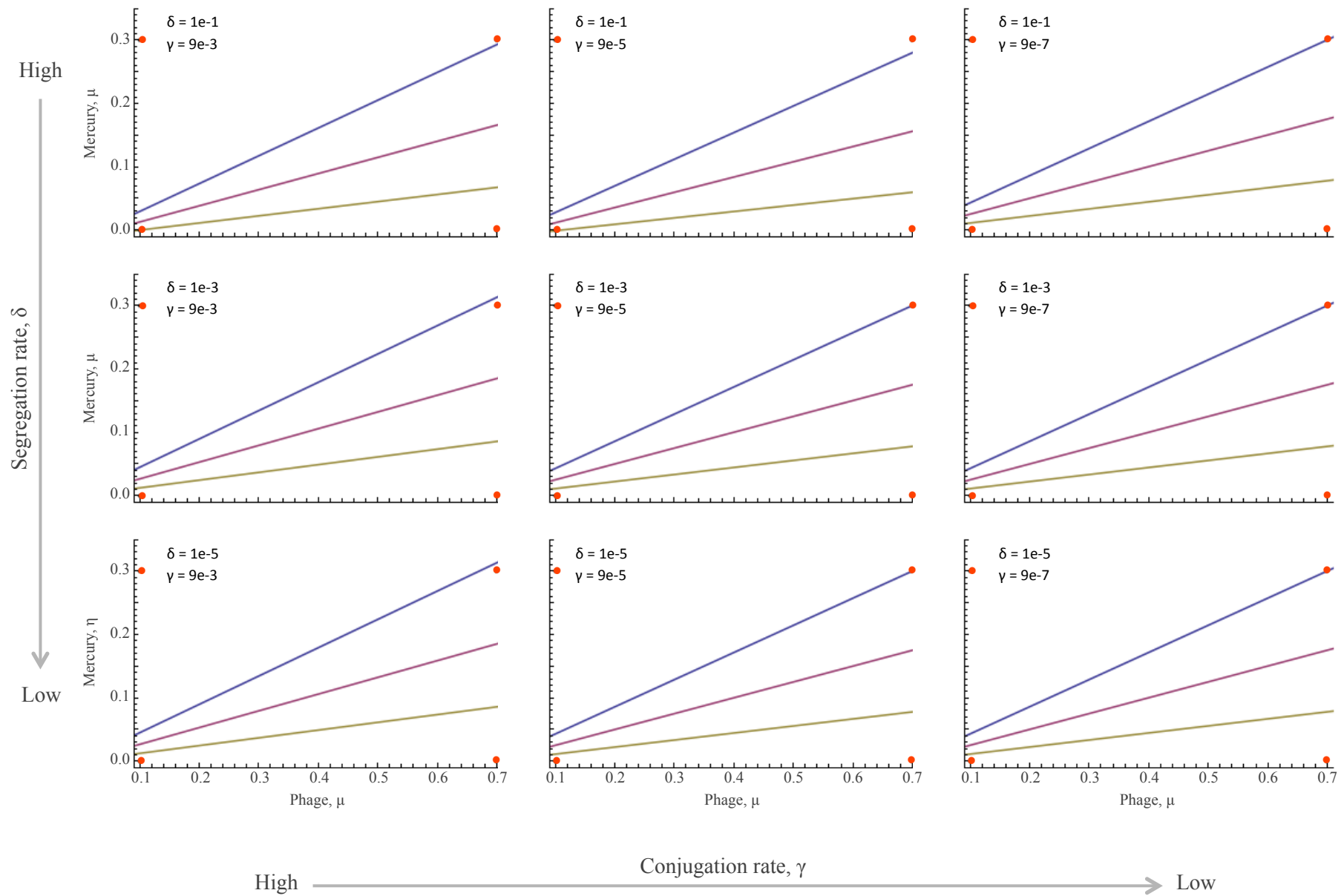

Supplement: Figure S4 — Exploration of the model across plasmid parameter space. The outcome of the model defined by equations 1 in the main text is shown, across a range of biologically plausible plasmid parameter values. The position of the line of transcritical bifurcations is plotted for segregation rates and conjugation rates 2 orders of magnitude above and below those approximated for the SBW25-pQBR103 system. The center plot therefore represents the parameters used in the model. Lines show the line of transcritical bifurcations at three levels of plasmid-containing growth rate (β): 0.7 (purple), 0.8 (red), and 0.9 (yellow). Red dots show the μ and η parameters sampled in the experiment. Despite movement in the position of the line, the qualitative predictions of the model are unchanged. Download [file mbo003152350sf4.pdf]

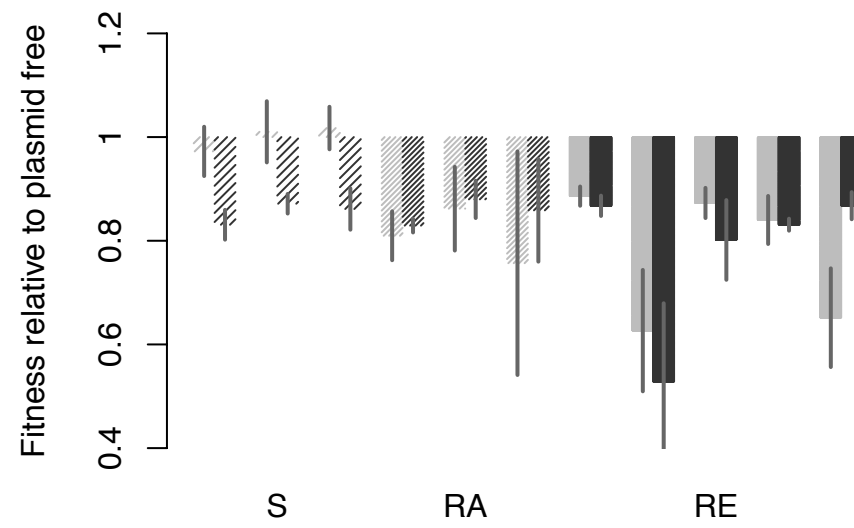

Supplement: Figure S7 — Fitness effects of phage resistance and plasmid carriage. Eight phage-resistant mutants were generated against a diverse set of phages (3 against the ancestral phage and 5 against phages taken from the evolved populations). The plasmid was then introduced to 3 sensitive and 8 resistant clones, and fitness was estimated based on competition with a marked plasmid-free ancestor. Sets of bars show mean fitness estimates (n = 3) for individual clones without (light) or with (dark) the plasmid. Shading shows phage sensitivity/resistance (sensitive, wide hash marks; resistant to ancestral phage, narrow hash marks; resistant to evolved phages, solid). Download [file mbo003152350sf7.pdf]
